# Evaluation of Genotype-Based Gene Expression Model Performance: A Cross-Framework and Cross-Dataset Study

**DOI:** 10.3390/genes12101531

**Published:** 2021-09-28

**Authors:** Vânia Tavares, Joana Monteiro, Evangelos Vassos, Jonathan Coleman, Diana Prata

**Affiliations:** 1 Instituto de Biofísica e Engenharia Biomédica, Faculdade de Ciências, Universidade de Lisboa, 1749-016 Lisboa, Portugal; vstavares@fc.ul.pt (V.T.); js.monteiro@campus.fct.unl.pt (J.M.); 2 Faculdade de Medicina, Universidade de Lisboa, 1649-028 Lisboa, Portugal; 3 Faculdade de Ciências e Tecnologia, Universidade Nova de Lisboa, 2829-516 Almada, Portugal; 4 Social, Genetic and Developmental Psychiatry Centre, Institute of Psychiatry, Psychology & Neuroscience, King’s College London, London SE5 8AF, UK; evangelos.vassos@kcl.ac.uk; 5 NIHR Maudsley Biomedical Research Centre, South London and Maudsley NHS Trust, London SE5 8AZ, UK; 6 Instituto Universitário de Lisboa (Iscte-IUL), CIS-Iscte, 1749-016 Lisboa, Portugal

**Keywords:** expression quantitative trait loci, transcriptome, gene expression, genome wide association study, polygenic score

## Abstract

Predicting gene expression from genotyped data is valuable for studying inaccessible tissues such as the brain. Herein we present eGenScore, a polygenic/poly-variation method, and compare it with PrediXcan, a method based on regularized linear regression using elastic nets. While both methods have the same purpose of predicting gene expression based on genotype, they carry important methodological differences. We compared the performance of expression quantitative trait loci (eQTL) models to predict gene expression in the frontal cortex, comparing across these frameworks (eGenScore vs. PrediXcan) and training datasets (BrainEAC, which is brain-specific, vs. GTEx, which has data across multiple tissues). In addition to internal five-fold cross-validation, we externally validated the gene expression models using the CommonMind Consortium database. Our results showed that (1) PrediXcan outperforms eGenScore regardless of the training database used; and (2) when using PrediXcan, the performance of the eQTL models in frontal cortex is higher when trained with GTEx than with BrainEAC.

## 1. Introduction

The emergence of genome-wide association studies (GWAS) has allowed the identification of associations between thousands of variants (mainly single nucleotide polymorphisms (SNP)) and susceptibility to complex diseases, such as schizophrenia [1]. However, there is still a gap between the variants and their functional role in the diseases’ etiologies, in particular in regards to SNPs [2]. Indeed, nearly 90% of these genetic variations occur in non-coding deoxyribonucleic acid (DNA) sequences, and only about 4–5% of plausibly causal variants in GWAS-associated regions are coding variants, which suggests that the main mechanism by which variation in these regions acts is not by altering protein structure. In comparison, about 50% of plausibly causal variants are expression quantitative trait loci (eQTL), suggesting moderation of gene expression is an important mechanism of action [3,4]. As such, it is crucial to consider and efficiently utilize variants correlated with gene expression, i.e., eQTL, to better understand the mechanisms behind the role of specific genes (especially if implicated by the hypothesis-free GWA approach) in intermediate or complex phenotypes [5].

The degree of expression of genes is typically inferred from the transcriptome, i.e., the messenger ribonucleic acid (mRNA) levels of the genes. One of the reasons for the delayed translation of transcriptome-wide association studies (TWAS) into clinical practice is that a gene expression profile is tissue-specific [6]. This is a crucial factor for a correct clinical interpretation of the eQTLs identified in the TWAS. However, in clinical practice, sampling invasiveness is the most important determinant for decision making regarding tissue sampling [7]. Indeed, measuring the expression of a given gene is invasive for many tissues, including the human brain, requiring postmortem sampling. Therefore, there is an urgent need for accurate statistical methods for the non-invasive estimation of gene expression in tissues where sampling presents more risks than the expected clinical benefit. Recently, efforts have been put forward to compile large-scale concomitant transcriptomic and genomic datasets, i.e., eQTL datasets, such as the Genotype-Tissue Expression (GTEx) project [8] across tissues, and the brain-specific Brain eQTL Almanac (BrainEAC) [9] and CommonMind Consortium (CMC) [10]. Using these emerging eQTL datasets, gene expression can be used as an intermediate molecular phenotype to potentially address the functional gap in GWAS findings and take a much needed step closer to understanding the underlying mechanisms and molecular pathways of complex disorders.

Recently, a gene-based method, PrediXcan, was developed and has been widely used to predict gene expression levels from SNPs [11,12,13,14]. In particular, using eQTL data and effect sizes determined through comprehensive eQTL analyses on reference transcriptome datasets, PrediXcan can predict expression levels for the whole transcriptome in multiple tissues [12]. Gamazon and colleagues [12] compared two methods for eQTL-based gene expression prediction: one based on traditional polygenic scoring [15] with one based on a regularized regression analysis using elastic net [16]; they showed that the latter yielded higher correlation between the observed and predicted gene expressions. However, the comparison was potentially biased, since (1) linkage disequilibrium (LD) between variations was not accounted for in the polygenic approach used; and (2) PrediXcan models do not account for individual missing genotypes; i.e., these are simply replaced by zero, thus assuming that that particular SNP makes the same contribution to the predicted gene expression level as a common homozygous genotype. The elastic net method consisted of (1) variable selection (i.e., selecting only SNPs that influence gene expression); and (2) handling highly correlated SNPs (i.e., those in high LD) by balancing their contribution to the variance in gene expression [12]. The polygenic method used consisted of (1) the selection of SNPs influencing gene expression by individually testing the association of each allele with gene expression through a linear regression; and (2) predicting gene expression as a weighted sum (taken from the individual linear regression analysis) of the SNP’s alleles showing an association with the observed gene expression below a significance threshold (i.e., *p* < single top SNP, 1 × 10^−4^, 0.001, 0.01, 0.05, 0.5 or 1). 

We herein tackled the two above-described limitations, i.e., the inaccurate polygenic method to which PrediXcan was originally compared and the passive incorporation of missing genotypes into the gene expression prediction. We did this by using an improved polygenic method to predict gene expression levels based on genome-wide genotypes, the eGenScore. In particular, we addressed the LD between gene expression-associated SNPs by filtering them out and the missing genotype issue by incorporating an adjustment factor to the weighted sum of SNP alleles based on the expected proportion of those alleles in a standardized population. After addressing the two issues above, we then aimed to compare our improved polygenic method with the PrediXcan elastic net method. Our second aim was to assess how training these tools with the most recent versions of each of the two main transcriptomic and genomic databases available, BrainEAC and GTEx, would affect their performance. To achieve both purposes, we trained eQTL models (which yield eQTL scores as a proxy of gene expression) with both frameworks, eGenScore and PrediXcan, and with each of the two databases, BrainEAC and GTEx, using transcriptomic data (i.e., gene expression levels) from the frontal cortex. We then compared the performance of the eQTL models across different frameworks (i.e., eGenScore vs. PrediXcan) and across databases (i.e., BrainEAC vs. GTEx), using an internal cross-validation approach and an external validation approach by applying the eQTL model to a third database from the CMC. Although the eGenScore method could theoretically be applied to any tissue type, herein we focused on the frontal cortex, as this tissue is the only one common to the three databases used in this study, i.e., BrainEAC, GTEx, and CMC.

## 2. Materials and Methods

**Figure 1:**
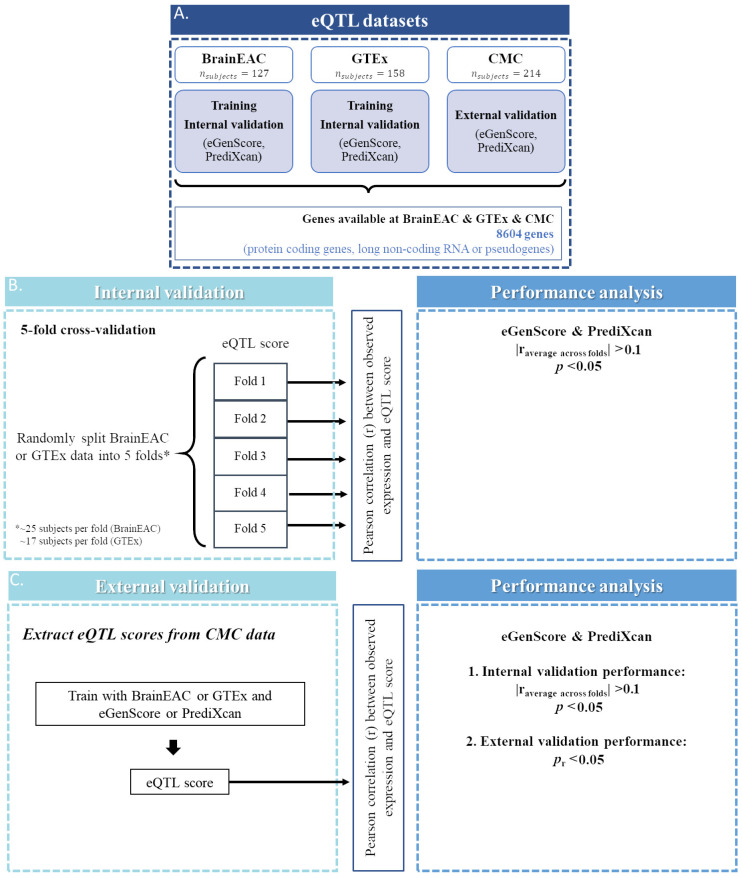
Representation of the steps taken for the selection of genes (**A**) for which an expression quantitative trait loci (eQTL) model was trained and validated, both internally (**B**) and externally (**C**). RNA: ribonucleic acid.

An overview of the datasets and methods used in this study is represented in Figure 1. All quality control procedures, described below, were performed by the database providers.

### 2.1. Genomic and Transcriptomic Datasets

***BrainEAC***. The BrainEAC dataset was used to train and internally validate eQTL models using eGenScore or PrediXcan. The dataset belongs to the UK Brain Expression Consortium (UKBEC) [9], was downloaded from the first version of the BrainEAC website (http://www.braineac.org/ (accessed on 19 May 2020)), and is composed of genome-wide genotypes and gene expression levels in the frontal cortex of 127 individuals. All samples and 5,712,227 SNPs have passed quality control (exclusion of individuals with non-European ancestry; samples with call rate < 95%; *p*-value for deviation from HWE < 10^−4^; genotyping call rate < 95%; poor post-imputation quality (R^2^ < 0.50); and minor allele frequency (MAF) < 5%). From these 127 individuals, gene expression levels for 25,501 genes (normalized using robust multi-array average, log_2_ transformed, and corrected for batch effects, sex, and brain bank) were provided by the BrainEAC dataset. Furthermore, the genomic data were mapped onto the human genome assembly GRCh37/hg19, the transcriptomic data were annotated according to NCBI Reference Sequence build 36, and, as for the databases GTEx and CMC and the 1000 Genomes data described below, only SNPs and exon-specific transcripts from chromosomes 1 to 22 were included in this study (i.e., sex chromosomes were excluded).

***GTEx***. The GTEx dataset (accessed from the GTEx Portal and dbGaP accession number phs000424.v8.p2, on 1 September 2020) was used to train and internally validate eQTL models using eGenScore or PrediXcan. The dataset is part of the Genotype Tissue Expression (GTEx) project conducted by the GTEx Consortium [17], and comprises whole genome sequencing and gene expression levels in the frontal cortex (Broadman area 9) of 158 individuals. All samples and 8,113,423 SNPs have passed quality control (exclusion of individuals with non-European ancestry; samples with call rate < 85%; *p*-value for deviation from HWE < 10^−8^; genotyping call rate < 85%; and minor allele frequency (MAF) < 1%). From these 158 individuals, gene expression levels for 17,354 genes in transcripts per million were provided by the GTEx dataset. Furthermore, the genomic data were mapped onto the human genome assembly GRCh38/hg38, and the transcriptomic data were mapped to GENCODE 26.

***CMC***. The CMC dataset (Release 1) was used to externally validate the eQTL models trained with eGenScore or PrediXcan using BrainEAC or GTEx. The dataset belongs to the CommonMind Consortium [10] and comprises genome-wide genotypes and gene expression levels in the frontal cortex (dorsolateral prefrontal cortex) of 214 individuals. All samples and 39,107,633 SNPs have passed quality control (exclusion of individuals with neuropsychiatric diseases—bipolar disorder, schizophrenia, or affective disorder—and with non-European ancestry; samples with call rate < 90%; *p*-value for deviation from HWE < 5 × 10^−5^; genotyping call rate < 98%). From these 214 individuals, gene expression levels for 15,478 genes in counts per million (normalized by scaling each sample’s read count to the total counts by gene, log_2_ transformed, and corrected for covariates using surrogate variables analysis) were provided by the CMC dataset. Furthermore, the genomic data were mapped onto the human genome assembly GRCh37/hg19, and the transcriptomic data were annotated to GENCODE 26.

***1000 Genomes***. The 1000 Genomes datasets (phase 3, October 2015, EUR panel) were used to compute LD and to adjust the weight of each SNP in the eQTL models trained with eGenScore using the BrainEAC dataset (1000 Genomes dataset 1) or the GTEx dataset (1000 Genomes dataset 2) [18]. Both datasets comprise genome-wide genotypes of individuals with European ancestry only (including Finnish). Dataset 1 comprises 78,089,780 SNPs mapped onto the human genome assembly GRCh37/hg19 of 503 individuals. Dataset 2 comprises 73,159,508 SNPs mapped onto the human genome assembly GRCh38/hg38 of 522 individuals.

### 2.2. Gene Overlap between Datasets

In this study we analyzed only genes that were labeled as protein coding, long non-coding RNA, or pseudogenes in GENCODE (v26, https://www.gencodegenes.org/human/release_26.html (accessed on 1 September 2020) and only if expression levels were available simultaneously in the BrainEAC, GTEx, and CMC datasets. Gene transcript IDs from BrainEAC and GTEx or CMC gene ensemble IDs were aligned using BioMart [19] by the following criteria: (1) the transcript ID and the gene ensemble ID should be from the same strand (i.e., positive or negative); and (2) when more than one transcript ID in the BrainEAC database correspond to the same gene ensemble ID in the GTEx or CMC database, the transcript ID with the largest overlap (in nucleotide base pairs) with the gene ensemble ID is chosen. Gene expression models were trained and validated internally and externally for 8604 genes with expression levels available in the BrainEAC, GTEx, and CMC databases (Figure 1A).

### 2.3. eQTL Model Training

**Figure 2:**
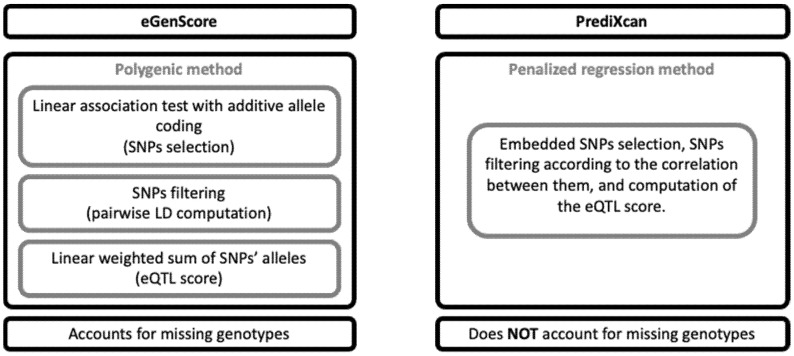
Comparison of the eGenScore’s and PrediXcan’s methodological features. LD: linkage disequilibrium; SNP: single nucleotide polymorphism.

An eQTL model for each gene was trained using each combination of the eGenScore and PrediXcan frameworks with the BrainEAC and GTEx datasets. The main differences between the eGenScore and the PrediXcan methods are represented in Figure 2. The first step, common to both frameworks, was to select SNPs located 1 million base pairs upstream and downstream of the gene location in the genome. The following steps are described separately for each framework below.

***eGenScore***. The association between the SNPs and the gene expression level was tested using linear regression and an additive allele coding (i.e., 0, 1, or 2 tested alleles) for each $SNP_{i}$ individually (Equation 1) and each $gene_{j}$ as implemented in Matrix eQTL [20].

(1) $$expression_{gene_{j}}=\beta_{i}\times SNP_{i}$$

SNPs nominally associated (*p* < 0.05) with gene expression were clumped using LD information from the 1000 Genomes dataset. In detail, the SNPs were first ordered by statistical significance (i.e., from the lowest to the highest *p*-value). Secondly, for every possible unique pair of SNPs, the LD was measured using the 1000 Genomes dataset. Thirdly, for each pair of SNPs in high LD (i.e., *r*^2^ > 0.3), the SNP with the lowest significance (i.e., the highest *p*-value) was excluded. Fourthly, the third step was iterated across all pairs of SNPs in high LD. Each SNP was weighted by the contribution of one tested allele of the SNP to the gene expression level (i.e., the β coefficients from the linear regression described above). The eQTL score, which represents the predicted gene expression, was computed for each $gene_{j}$ as the weighted sum of each $SNP_{i}$’s tested alleles adjusted to the expected proportion of those alleles in a standardized population (i.e., the 1000 Genomes dataset) (Equations (2) and (3)). For each $SNP_{i}$, this adjustment centers the expected contribution of the $SNP_{i}$ to the $eQTL\;score_{gene_{j}}$ at zero. If the genotype of $SNP_{i}$ is missing in an individual ($CalledSNP_{i}=0$), the contribution of $SNP_{i}$ to $eQTL\;score_{gene_{j}}$ is also set to zero. In this way, the contribution of a missing SNP to the eQTL score is equal to its expected contribution in the standardized population [21]. Furthermore, some examples of eQTL score computation are provided in Supplementary Material, Tables S1 and S2.

(2) $$eQTL\;score_{gene_{j}}=\underset{i}{\sum }\left(\left(\beta_{i}\times SNP_{i}-adjustment\;factor_{i}\right)\times CalledSNP_{i}\right)$$

where $CalledSNP_{i}=\{\begin{array}{c} 1,\;\mathrm{if}\;\mathrm{the}\;\mathrm{genotype}\;\mathrm{of}\;SNP_{i}\;\mathrm{is}\;\mathrm{present}\\ 0,\;\mathrm{if}\;\mathrm{the}\;\mathrm{genotype}\;\mathrm{of}\;SNP_{i}\;\mathrm{is}\;\mathrm{missing} \end{array}$.

(3) $$adjustment\;factor_{i}=\beta_{i}\times Proportion_{1\;ref\;allele;\;i}+2\times \beta_{i}\times Proportion_{2\;ref\;allele;\;i}$$

***PrediXcan***. The PrediXcan framework combines all the SNP selection, SNP LD exclusion, and gene expression prediction steps as described in the eGenScore section into one single step by using regularized linear regression methods [12]. In detail, gene expression is predicted by applying an elastic net regression to the original set of SNPs within the gene region (i.e., 1 million base pairs up- and downstream). Elastic net uses L1 and L2 penalties from least absolute shrinkage and selection operator (LASSO) and ridge regression, respectively, which allows the selection of a set of uncorrelated (i.e., sparse) SNPs [16]. Furthermore, the elastic net regression analysis was conducted using the R package glmnet [22] with α = 0.5. Herein, we used the gene expression models that were trained with the GTEx dataset and that are publicly available at PredictDB Data Repository (http://predictdb.org/ (accessed on 1 September 2020)). Additionally, we trained the gene expression models using the BrainEAC dataset, using the same specifications as those used with the GTEx database [12].

### 2.4. Internal Validation

The eQTL models were internally validated using a 5-fold cross-validation approach. In each iteration, the following measures were computed for each gene using the hold-out fold: (1) the Pearson correlation coefficient (*r*) between the observed gene expression and the eQTL score; and (2) the *p*-value corresponding to the null hypothesis of no correlation between the observed gene expression and the eQTL score. Then, as an overall performance measure of the gene expression model, the Pearson correlation coefficient was averaged across the 5 folds (*r_avg_*) and squared (*r_avg_*^2^). Furthermore, the *r_avg_*^2^ is herein interpreted as the variance in the observed gene expression levels that can be explained by the eQTL score (i.e., the predicted gene expression levels). The global *p*-value was computed using Fisher’s method [23], which was previously used by the authors of PrediXcan [12]. Furthermore, the models were considered significant if the averaged correlation between the observed gene expression and the eQTL score was statistically significant (i.e., Fisher’s *p*-value < 0.05) and of at least small size (i.e., |*r_avg_*| > 0.1). These performance measures were extracted for all models trained with eGenScore and the BrainEAC or GTEx dataset and PrediXcan and the BrainEAC dataset. They were already available for models trained with PrediXcan and GTEx (at the PredictDB Data Repository).

### 2.5. External Validation

The eQTL models which were shown to be significant at the internal validation were externally validated using the CMC dataset. The external validation performance was assessed by computing the Pearson correlation coefficient (*r*) between the observed gene expression and the eQTL score in the CMC dataset and considered statistically significant if the *p*-value corresponding to the null hypothesis of no correlation between the observed gene expression and the eQTL score was below 0.05. We additionally calculated the squared Pearson correlation coefficient (*r*^2^). These performance measures were extracted for all models trained with eGenScore or PrediXcan and the BrainEAC or GTEx dataset and PrediXcan and the BrainEAC dataset.

### 2.6. Performance Comparison between eQTL Models

The squared averaged Pearson correlation coefficient (i.e., *r_avg_*^2^) and the squared Pearson correlation coefficient (i.e., *r*^2^) of the internal and external validations, respectively, were compared across training datasets (BrainEAC vs. GTEx) and across frameworks (i.e., eGenScore vs. PrediXcan) for the genes whose models were significant in the tested combinations (i.e., BrainEAC vs. GTEx or eGenScore vs. PrediXcan) using a two-tailed paired *t*-test. Cohen’s *d* was computed as the effect size of the difference, and its interpretation was performed using Kristoffer Magnusson’s web tool (Interpreting Cohen’s d effect size, https://rpsychologist.com/d3/cohend/ (accessed on 5 April 2021)).

## 3. Results

### 3.1. eQTL Models Significant in Each Method

**Figure 3:**
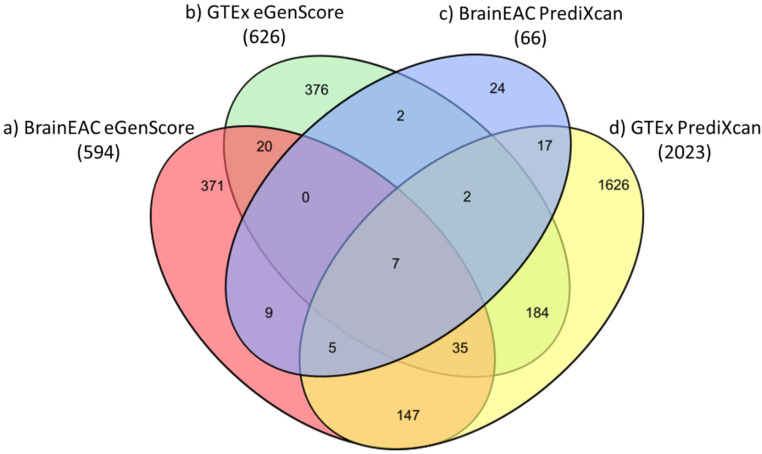
Venn diagram representing the number of genes for which an eQTL model trained with (a) BrainEAC and eGenScore (orange); (b) GTEx and eGenScore (green); (c) BrainEAC and PrediXcan (blue); or (d) GTEx and PrediXcan (yellow) was found to be statistically significant during the internal validation (i.e., |*r_avg_*| > 0.1 and Fisher’s *p*-value < 0.05).

The proportion of eQTL models (i.e., of genes) shown to be statistically significant (i.e., |*r_avg_*| > 0.1 and Fisher’s *p*-value < 0.05) was highest when they were trained with the PrediXcan framework and the GTEx database (23.5%; 2023 out of 8604 genes) and, in descending order, was followed by models trained with (1) eGenScore and GTEx (7.3%; 626 out of 8604 genes); (2) eGenScore and BrainEAC (6.9%; 594 out of 8604 genes); and (3) PrediXcan and BrainEAC (0.8%; 66 out of 8604) (Figure 3).

### 3.2. eQTL Model’s Internal Validation

**Figure 4:**
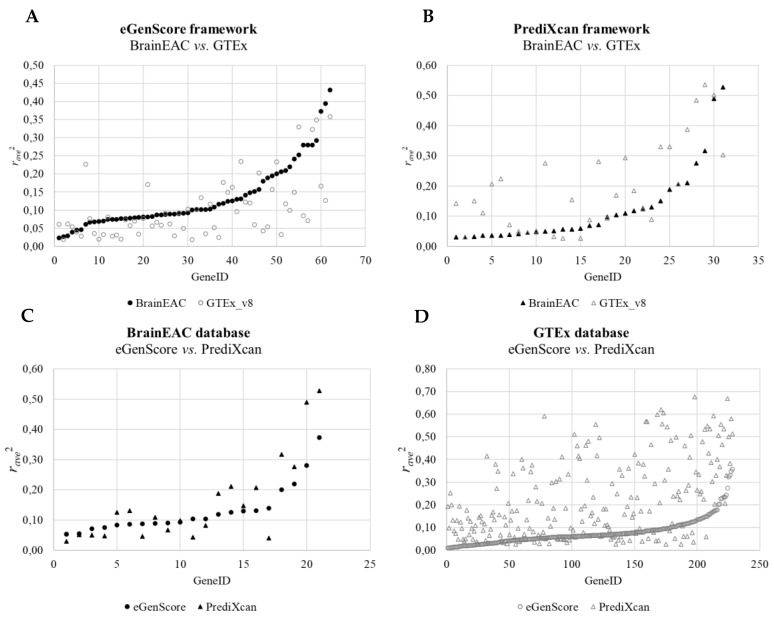
Comparison of the squared averaged Pearson correlation coefficient (*r_avg_*^2^) between the predicted and observed gene expressions during the internal cross-validation across databases (i.e., BrainEAC vs. GTEx) using the eGenScore (**A**) or the PrediXcan (**B**) framework and across frameworks (i.e., eGenScore vs. PrediXcan) using the BrainEAC (**C**) or GTEx (**D**) dataset. The model’s performance is represented with a filled black or hollow gray marker if trained with the BrainEAC or GTEx database, respectively, and with a circle or triangle if trained with the eGenScore or PrediXcan framework, respectively.

When comparing the internal validation performance of the gene expression models across databases, the squared averaged Pearson correlation coefficient between the observed gene expression and the eQTL score was statistically different between BrainEAC and GTEx using both frameworks (eGenScore, *p* = 0.003, or PrediXcan, *p* = 0.001) (Table 1 and Figure 4). Furthermore, the performance was shown to be higher for BrainEAC when using eGenScore and for GTEx when using PrediXcan. When comparing the eQTL models’ performance across frameworks, models trained with PrediXcan showed better performance than the ones trained with eGenScore (*p* < 0.001) but only when using the GTEx database (Table 1 and Figure 4).

### 3.3. eQTL Model’s External Validation

Across databases, the external validation performance of the eQTL models (i.e., when applied to the CMC database) was shown to be higher when trained with the BrainEAC than with the GTEx database but only when using the eGenScore framework (*p* = 0.015) (Table 2). No statistically significant difference in the external validation performance of the eQTL models was found when models were trained with the BrainEAC or GTEx database and PrediXcan framework (Table 2). Across frameworks, the performance of the gene expression models was shown to be higher for PrediXcan compared to eGenScore when using BrainEAC (*p* = 0.018) or GTEx (*p* < 0.001) datasets (Table 2).

## 4. Discussion

We herein presented eGenScore, a polygenic score-based method to predict gene expression levels from genotypes. In a previous paper, the elastic net-based framework PrediXcan was shown to outperform a polygenic score-based method [12]. However, that polygenic score-based method was methodologically limited; in comparison to it, eGenScore better handles the following two issues: (1) the LD between SNPs shown to be individually associated with gene expression; and (2) missing genotypes. We compared the performance of frontal cortex-specific gene expression models trained with different frameworks, eGenScore vs. PrediXcan, as well as with different datasets, BrainEAC vs. GTEx, after both internal and external validation steps.

Overall, our results confirm that elastic net-based methods are superior to polygenic score-based methods for the prediction of gene expression based on eQTL genotypes. PrediXcan predicted gene expression levels with a higher performance than eGenScore regardless of the database (i.e., BrainEAC or GTEx) used for model training. Indeed, the observed difference in the internal validation performance between frameworks when using the GTEx database corresponded to a large effect size, with roughly 82% of the gene expression models showing higher performance when trained with PrediXcan than with eGenScore (i.e., 186 out of 228 genes; Cohen’s *d* = 0.92). This effect was enlarged when the gene expression models were applied to an external database (i.e., CMC), with 93% of the gene expression models showing a higher correlation between the observed and predicted gene expressions in the CMC database when trained with PrediXcan than with eGenScore (i.e., 108 out of 116 genes; Cohen’s *d* = 1.46). However, the frameworks differed in their best training dataset; models trained with BrainEAC outperformed those trained with GTEx when the eGenScore framework was used, whereas the opposite was observed when the PrediXcan framework was used. The effect of the training database on performance was shown to be higher for the PrediXcan framework, with 74% of the gene expression models showing higher internal performance when trained with GTEx (i.e., 22 out of 31 genes; Cohen’s *d* = 0.65). This higher dependence on the training dataset may compromise an assumption of generalizability of PrediXcan across training sets.

Given that PrediXcan was shown to be a better framework for predicting gene expression levels than eGenScore, our results suggest that GTEx should be used as the training database for these gene expression models. When compared with BrainEAC, GTEx is a more comprehensive transcriptomic and genomic database with a slightly larger sample size for brain gene expression and uses whole-genome sequence data and gene expression data (compared to gene expression array data employed in BrainEAC). This different source of gene expression data may explain the lack of concordance between eQTL models derived using the same framework in different datasets and the poorer performance of PrediXcan in BrainEAC compared to GTEx. The challenges of obtaining gene expression data in brain tissues may necessitate a variety of approaches to measuring brain gene expression. As such, a valuable direction for future research may be to improve the generalizability of elastic net-based frameworks such as PrediXcan to work effectively across different sources of gene expression data. Indeed, this may be a particularly important step towards a universal, non-invasive, statistical estimator of tissue-specific gene expression, a very important tool for an effective translation of TWAS into clinical practice [7].

## 5. Limitations

Our study had several limitations that need to be addressed. Firstly, the gene expression data of the BrainEAC and GTEx databases were annotated to different human genome assemblies, which hindered the exact correspondence between the transcriptomic data of the two databases and, therefore, narrowed the number of possible genes that we could analyze. Secondly, although we restricted the comparisons of the performance of gene expression models to genes expressed in the frontal cortex, in fact, the brain samples from which the transcriptomic data were extracted across databases were not exactly from the same brain location. It is reasonable to expect that the expression level of a given gene might slightly vary depending on which exact location in the brain it is taken from. Therefore, the comparison of model performance across datasets might be influenced by this factor. Thirdly, both eGenScore and PrediXcan methods rely on genotype–gene expression association data (such as are provided in the BrainEAC and GTEx databases) and, therefore, are only valid for the age interval of the sample used in these databases. They cannot account for the epigenetic effects on gene expression across the lifespan.

## 6. Conclusions

In this study, we compared the performance of eQTL models trained with: (1) different frameworks—eGenScore, a novel (introduced for the first time herein) and improved polygenic method that, compared with the original polygenic method presented along with PrediXcan, addresses high LD between SNPs and handles individual missing genotypes; and PrediXcan, a previously published and regularized linear regression method (i.e., elastic net)—; and (2) different training datasets—BrainEAC and GTEx. Taken together, our results show that: (1) PrediXcan outperforms eGenScore regardless of the training database that is used (i.e., BrainEAC or GTEx); and (2) GTEx yields eQTL models with a higher performance than BrainEAC when using PrediXcan. Therefore, we encourage the use of models trained with the GTEx database and using the PrediXcan framework when predicting gene expression from genotype data.

## Figures and Tables

**Table 1:** Comparison of the gene expression models’ internal validation performance (i.e., the squared averaged Pearson correlation coefficient between the predicted and observed gene expressions) across datasets (i.e., BrainEAC vs. GTEx) and across frameworks (i.e., eGenScore vs. PrediXcan).

Comparison	df, *t*	*p*	Cohen’s *d*
eGenScore framework (BrainEAC vs. GTEx)	61, 3.10	0.003 **	0.39
PrediXcan framework (BrainEAC vs. GTEx)	30, −3.63	0.001 ***	0.65
BrainEAC dataset (eGenScore vs. PrediXcan)	20, −1.79	0.088	0.39
GTEx dataset (eGenScore vs. PrediXcan)	227, −13.86	<0.001 ***	0.92

Only genes with a significant model (i.e., with an absolute averaged Pearson correlation coefficient between the predicted and observed gene expressions above 0.1 and a Fisher’s *p*-value below 0.05) were considered for this comparison. A two-sided paired-sample *t*-test was conducted and considered statistically significant at a *p*-value < 0.05. df: degrees of freedom (i.e., number of genes for which there was a significant model minus one). **: *p* < 0.01; ***: *p* < 0.001; *t*: t-statistic.

**Table 2:** Comparison of the gene expression models’ external validation performance (i.e., the squared Pearson correlation coefficient between the predicted and observed gene expressions in the CMC dataset) across datasets (i.e., BrainEAC vs. GTEx) and across frameworks (i.e., eGenScore vs. PrediXcan).

Comparison	df, *t*	*p*	Cohen’s *d*
eGenScore framework (BrainEAC vs. GTEx)	33, 2.57	0.015 *	0.44
PrediXcan framework (BrainEAC vs. GTEx)	15, −2.04	0.060	0.51
BrainEAC dataset (eGenScore vs. PrediXcan)	8, −2.95	0.018 *	0.98
GTEx dataset (eGenScore *vs.* PrediXcan)	115, −15.76	<0.001 ***	1.46

Only genes with a statistically significant Pearson correlation coefficient between the predicted and observed gene expressions in the CMC dataset (*p*-value < 0.05) were considered for this comparison. A two-sided paired-sample *t*-test was conducted and considered statistically significant at a *p*-value < 0.05. df: degrees of freedom (i.e., number of genes for which there was a model whose predicted expression correlated significantly with the observed expression of that gene minus one). *: *p* < 0.05; ***: *p* < 0.001; *t*: t-statistic.

## Data Availability

Data is available upon request to the authors.

## Supplementary Materials

The following are available online at https://www.mdpi.com/article/10.3390/genes12101531/s1, Table S1: β values, adjustment factor, and coding alleles for the list of SNPs to be included in the eQTL score model for the neuregulin 4 gene (*NRG4*); Table S2: Examples of how the eQTL score is computed. In detail, eQTL score was computed for four hypothetical individuals.
